# Online modules to improve health professionals’ end-of-life law knowledge and confidence: a pre-post survey study

**DOI:** 10.1186/s12904-023-01290-6

**Published:** 2023-10-31

**Authors:** Rachel Feeney, Lindy Willmott, Penny Neller, Shih-Ning Then, Patsy Yates, Ben White

**Affiliations:** 1 Australian Centre for Health Law Research, Queensland University of Technology, Brisbane, QLD Australia; 2 Faculty of Health, Queensland University of Technology, Brisbane, QLD Australia

**Keywords:** Allied health occupations, Health legislation, Medicine, Nursing, Online learning, Palliative care, Survey methods

## Abstract

**Background:**

Health professionals and medical students have knowledge gaps about the law that governs end-of-life decision-making. There is a lack of dedicated training on end-of-life law and corresponding research on the impact of this type of training.

**Objective:**

To examine the impact of online training modules on key concepts of end-of-life law on Australian health professionals’ legal knowledge and their self-reported confidence in applying the law in practice.

**Methods:**

Online pre- and post-training surveys were completed by training participants. The optional surveys collected demographic data, directly assessed legal knowledge and measured self-reported confidence in applying the law in clinical practice, before and after training.

**Results:**

Survey response rates were 66% (pre-training) and 12% (post-training). The final sample for analysis (*n* = 136 participants with matched pre- and post-training surveys), included nurses, doctors, allied health professionals, medical students and a small number of non-health professionals. Following completion of the online training modules, legal knowledge scores significantly increased overall and across each domain of end-of-life law. Participants were also more confident in applying the law in practice after training (median = 3.0, confident) than before training (median = 2.0, not confident).

**Conclusions:**

This study found that completion of online training modules on end-of-life law increased Australian health professionals’ legal knowledge and self-reported confidence in applying the law in clinical practice. Participants demonstrated some remaining knowledge gaps after training, suggesting that the training, while effective, should be undertaken as part of ongoing education on end-of-life law. Future research should examine longer term outcomes and impacts of the training.

## Introduction

End-of-life law governs clinical decision-making at the end of life as well as the provision of palliative care and advance care planning [1]. As a result, health professionals play critical legal as well as clinical roles when providing end-of-life care [2]. In Australia (the site of the current study) common legal roles played by a broad range of health professionals include recognising or determining whether a person has capacity for medical treatment decision-making, knowing when it is lawful to withhold or withdraw treatment and identifying when treatment is likely to be futile or non-beneficial [3]. Where a person does not have capacity, health professionals need to identify the person’s substitute decision-maker or decide whether to follow a person’s advance care directive (where there is one available) [4].

By regulating clinical practice, end-of-life law safeguards the interests of patients and protects health professionals who act within the boundaries of the law [5–7]. Yet there is evidence that health professionals have knowledge gaps in this area [5, 8–11]. Further, education and training on end-of-life law (from undergraduate university through to continuing professional development) is limited [5, 12, 13].

High-quality, relevant education and training is essential to the growth and development of the health workforce, including those working in end-of-life care [14]. There is some research which examines the benefits of clinical training on end-of-life care for health professionals and health professional students [15–20]. Studies have generally found that training enhances health professionals’ knowledge, confidence and attitudes in relation to end-of-life care [17–22]. Evidence of the impact of training on clinical practice is less clear. While some studies have shown promising results, [23–28] further research is needed given common methodological limitations of many previous studies, including a lack of baseline measures taken prior to education or training, and/or a reliance on self-report measures rather than objective assessments for examining training outcomes [17–20].

There is also a dearth of research examining outcomes of dedicated training on end-of-life law. A small number of studies have evaluated training programs that include content on law and advance care planning; [29–31] however they have not separately examined training outcomes related to legal rather than clinical aspects of the training. One recent health law study [32] examined the impact of a one hour legal education session on consent law (relevant but not specific to the end-of-life context). It reported significant improvements in health professionals’ knowledge after education, however analysis involved comparisons of aggregated pre- and post-education data rather than examination of within-person changes.

The current study addresses existing gaps in the research literature by examining the impact on health professionals’ knowledge of an Australian end-of-life law training program. This training focuses exclusively on legal content, covering the key concepts of end-of-life law in all States and Territories. The curriculum is tailored to a broad range of health professionals (doctors, nurses, allied and other health professionals) and medical students, reflecting the fact that good end-of-life care is delivered by a multi-disciplinary team [33]. Pre- and post-training surveys measure multiple training outcomes including health professionals’ legal knowledge and their confidence in applying the law in practice. Legal knowledge is directly assessed, providing a better proxy for clinical competence than self-reported knowledge. The study extends our earlier cross-sectional work examining Australian health professionals’ baseline knowledge of end-of-life law and predictors of legal knowledge [5].

## Methods

### Study design

End of Life Law for Clinicians (ELLC) is a national training program on end-of-life law funded by the Australian government. ELLC provides free training for Australian medical practitioners, medical students, nurses and allied and other health professionals, available online at ellc.edu.au [34]. It aims to improve health professionals’ knowledge and awareness of the Australian law at end of life to support their delivery of quality end-of-life and palliative care. At the time of writing, ELLC comprises 12 online training modules (the focus of this study) addressing different domains of end-of-life law (see Table 1). The modules contain interactive exercises, legal cases, clinical case studies, vignettes, self-assessment quizzes, and further readings to promote reflective learning. Participants can complete the entire course, or only some modules. Each Australian State and Territory has its own end-of-life laws that, while similar, have some key differences. Therefore the modules cover the fundamental concepts of end-of-life law in Australia with some exploration of jurisdictional differences. The modules are supported by the website End of Life Law in Australia, [35] which provides more detailed and complementary legal information about specific end of life laws in each Australian State and Territory for those who want it. ELLC also includes case-based workshops and webinars delivered nationally (data not reported here). Workshops and webinars are tailored for different clinical audiences and jurisdictions and are delivered in a co-presenter model with an ELLC team member and a clinical co-presenter. To date, 50 workshops have been delivered to over 4100 attendees.

This paper reports on the analysis of data collected as part of the online training’s pre-and post-training surveys. Analysis was undertaken to obtain an assessment of training impact in two areas: participant’s knowledge of end-of-life law and their self-reported confidence in applying end-of-life law in practice. Data were collected between 31 January 2019 and 31 October 2022.

**Table 1:** Online training modules

1. The role of law in end of life care
2. Capacity and consent to medical treatment
3. Withholding and withdrawing life-sustaining medical treatment
4. Advance care planning and advance care directives
5. Substitute decision-making for medical treatment
6. Legal protection for administering pain and symptom relief
7. Children and end of life decision-making
8. Futile or non-beneficial treatment
9. Emergency treatment for adults
10. Managing conflict
11. Voluntary assisted dying
12. Aboriginal and/or Torres Strait Islander peoples and end of life law

### Sample

The training (and embedded surveys) was initially promoted to Australian doctors (medical specialists including GPs, trainee specialists, interns, registrars) and medical students by engaging relevant stakeholders across the palliative care and health sectors. Promotion was later expanded to include nurses and allied and other health professionals (including Aboriginal and Torres Strait Islander health workers/practitioners and paramedics). The training was, however, publicly available and therefore was also accessed by a small number of non-health professionals. Training participants were largely a self-selecting group, although some medical students were required to complete modules as part of their degree program.

### Survey instrument

The survey development process and content of the pre-training survey has been described elsewhere [5]. The post-training survey contained nine sections: demographics (three items); modules completed (one item); perspectives on law and end-of-life decision-making (10 items); self-perceived knowledge of end-of-life law (one item); actual knowledge of law (10 items); experience in applying the law in practice (seven items); recent continuing professional development (CPD) training in end-of life-law (one item); self-assessed confidence in the ability to apply the law in practice (one item); feedback on the ELLC training (10 items).

This paper focuses on participants’ actual knowledge of law and self-assessed confidence in their ability to apply the law in practice (before and after training). Ten questions in the pre- and post-training surveys assessed legal knowledge across the following areas of law: capacity and consent (three questions), withholding and withdrawing life sustaining treatment (three questions), advance care directives (two questions), substitute decision-making (one question) and legal protection for administering pain and symptom relief (one question). Survey questions were based on content from the first six ‘foundational’ modules (see Table 1). Response options were true, false or ‘I don’t know’ in relation to participants’ relevant State or Territory law (based on participants’ work postcode). Participants received a score of one for each correct answer (‘I don’t know’ marked as incorrect), resulting in a score of 0–10. Participants also rated their pre and post-training confidence in applying the law in practice (‘not at all confident’; ‘not confident’; ‘confident’; ‘very confident’).

### Data collection

Both surveys were optional and completed online via Key Survey. Training participants were asked to complete the pre-training survey before commencing training. Survey completion was promoted on the training website homepage, in the first training module, and via a targeted reminder email to survey non-respondents. A link to the post-training survey form was distributed via email to participants who completed six or more online modules. These participants were considered to have sufficient exposure to the training to have an increased knowledge of law and be able to provide feedback on the training.

### Data analysis

Data on sample characteristics, knowledge of end-of-life law and confidence in applying the law were analysed using SPSS 28. Statistical significance was set at *P* ≤ .05. Following initial descriptive analysis, differences in pre and post-training legal knowledge (mean scores and proportion of participants scoring ≥ 6/10) were examined using paired-samples t-tests. Differences in pre and post-training confidence in applying the law were examined using the Wilcoxon signed-rank test.

## Results

### Sample characteristics

Response rate was 66% for the pre-training survey (4898/7370 total training registrants) and 12% for the post-training survey (174/1447 post-training surveys distributed). The final sample for analysis was 136 cases (78% of the 174 post-training survey participants). Participants were included in the analysis if they had matched pre- and post-training surveys.

The sample comprised nurses (45%), doctors (34%), allied and other health professionals (12%), medical students (7%) and non-health professionals (2%) (Table 2). Participants were mostly female (74%) and aged between 35 and 64 years (82%). Most common work settings were hospitals (46%) and residential aged care facilities (10%); 11% were students with no current work setting. Participants reported an average of 17 years of clinical practice (range 0–52 years). Most participants (82%) reported that they had not undertaken any education or training about end-of-life law other than ELLC in the last 12 months.

**Table 2:** Sample characteristics

Variable	Frequency	Percent
**Total**	136	100.0
**Profession**		
Nurse ^a^	61	44.9
Doctor	46	33.8
Allied health ^b^	16	11.8
Medical student	10	7.4
Non-health professional	3	2.2
**Gender**		
Female	100	73.5
Male	36	26.5
**Age**		
< 25 years	4	2.9
25–34 years	18	13.2
35–44 years	32	23.5
45–54 years	29	21.3
55–64 years	51	37.5
65 years and over	2	1.5
**State**		
Queensland	40	29.4
Victoria	39	28.7
New South Wales	34	25.0
Western Australia	11	8.1
South Australia	9	6.6
Australian Capital Territory	2	1.5
Tasmania	1	0.7
Northern Territory	0	0.0
**Main work setting**		
Hospital	63	46.3
Residential aged care facility	14	10.3
Specialist palliative care service	11	8.1
General practice	10	7.4
Private practice	8	5.9
Community health	7	5.1
Other	12	8.8
Not applicable (medical/nursing student)	11	8.1

^a^Includes one nursing student. ^b^Includes paramedics, social workers, speech pathologists, occupational therapists, psychologists, physiotherapists, Indigenous Hospital Liaison Officers, radiographers, audiometrists and music therapists

### Knowledge of end-of-life law

Mean scores from the pre-training and post-training surveys showed a significant increase in participants’ legal knowledge following completion of the ELLC modules [t(135) = -20.4, *p* < .001]. The pre-training mean was 5.65 (out of 10, SD 1.89) and the post-training mean was 7.92 (out of 10, SD 1.32) (Table 3). Mean knowledge scores increased by an average of 2.3 after training. Consistently, the proportion of participants who correctly answered most questions (≥ 6) after training (*n* = 129, 94.9%) was significantly higher than before training (*n* = 63, 46.3%) [t(135) = -11.3, *p* < .001]. This significant increase in knowledge occurred across all domains of end-of-life law assessed (i.e. capacity and consent, withholding and withdrawing life-sustaining treatment, advance care directives, substitute decision-making, pain and symptom relief).

**Table 3:** Pre- and post-training knowledge of end-of-life law (*n* = 136)

	Pre-training		Post-training		Mean difference	t	p
	Mean	SD	Mean	SD			
Total score	5.65	1.89	7.92	1.32	2.26	-20.39	< 0.001
Capacity and consent (3 questions)	1.32	0.72	1.51	0.80	0.20	-2.42	0.017
Withholding and withdrawing life-sustaining treatment (3 questions)	1.86	1.03	2.54	0.64	0.68	-7.25	< 0.001
Advance care directives (2 questions)	1.32	0.69	1.61	0.55	0.29	-4.24	< 0.001
Substitute decision-making (1 question)	0.92	0.27	0.99	0.09	0.07	-2.97	0.004
Pain and symptom relief (1 question)	0.24	0.43	0.35	0.48	0.12	-2.09	0.038

### Confidence in applying the law

On average, training participants were more confident in applying the law in practice after training (median = 3.0, confident) than before training (median = 2.0, not confident). This increase of 1 on 4 point scale was statistically significant (T = 3570, z = -8.66, *p* < .001). Interestingly, even after the training, relatively few participants (7%) reported feeling very confident in their ability to apply the law in practice.

## Discussion

Legal knowledge is an important component of health professionals’ expertise [13] This study examined outcomes of a unique training program on end-of-life law in Australia, delivery of which fills an identified gap in currently available training and education. It presents new knowledge about the effectiveness of training on end-of-life law in improving health professionals’ legal knowledge and confidence in applying the law in clinical practice. Following completion of the ELLC online training modules, participants demonstrated significantly better knowledge of end-of-life law (overall and in each legal domain assessed). Participants also reported significantly enhanced confidence in applying the law in practice. These findings of enhanced knowledge and confidence after training are consistent with the literature on clinical training on end-of-life care [16–19]. Arguably, improvements were not only statistically significant but large enough to be meaningful in a clinical setting. On average, participants demonstrated moderate legal knowledge (5.7 out of 10 correct) before training and good legal knowledge after it (7.9 out of 10 correct). Further, participants were, on average, not confident in their ability to apply the law in practice before training but were confident after training. Because most participants (82%) had not undertaken any other recent education or training about end-of-life law, it is likely that these results are due to completion of the ELLC modules. This study therefore supports the utility of this training in helping to address gaps in health professionals’ knowledge of end-of-life law and their confidence to engage with and apply it.

Most participants demonstrated post-training knowledge gaps, with only 9% correctly answering all knowledge questions. This finding is consistent with a study of legal education on consent law, [32] which also reported persistent errors after education. Additionally, after our training, relatively few participants (7%) reported being very confident in their ability to correctly apply the law in practice. This may reflect the complexity of end-of-life law in Australia [7]. Thus, while the training demonstrated positive outcomes overall, health professionals who require a very high level of proficiency in end-of-life law (including doctors, who often carry the legal responsibility for end-of-life decision-making), may benefit from ongoing education and training in this area. This suggestion is supported by our earlier paper on this training, [5] which found that participants’ other recent CPD training significantly improved participants’ legal knowledge.

Consistent with the broader literature, [18] this study did not examine the impact of training on clinical practice. Participants’ feedback on the training (not reported here) indicated that they perceived a close relationship between training content and their work duties, which is a prerequisite for transfer of knowledge from training to clinical practice [36]. Future research should examine longer term outcomes and impacts of the training. This may include the extent to which participants apply the training in their clinical practice and whether training has a measurable impact on workplace behaviours including clinical decision-making and quality and lawfulness of care.

### Strengths and limitations

This study extends the research literature and addresses existing research gaps by examining the impact of an Australian training program for health professionals and medical students on end-of-life law. There is currently very little research on the impact of targeted training on end-of-life law on these groups. Methodological strengths of the study include use of baseline measures taken prior to training, direct assessment of legal knowledge (a key outcome measure), and examination of within-person changes.

Some limitations apply to the study. Analyses were based on a small sample as the post-training survey had a low response rate and not all pre-training participants submitted a post-training survey. Participants may represent a highly motivated and interested subset of the online training participants and broader health professional population, and this may affect generalisability of the study results. Because of the small sample size, predictors of changes in legal knowledge could not be explored. The study design did not allow for a control group as the training aimed to reach the largest possible target audience.

## Conclusion

This study of Australian health professionals and medical students found that completion of online training modules on end-of-life law successfully increased participants’ legal knowledge and self-reported confidence in applying the law in clinical practice. Importantly, increases in knowledge were observed in all domains of end-of-life law assessed. However, participants demonstrated some remaining knowledge gaps post training, and relatively few reported being at the highest confidence level (very confident) in terms of their ability to correctly apply the law in their clinical practice. This finding suggests that the training, while effective, should be undertaken as part of ongoing education and training in end-of-life law (e.g. through integrating the modules into university curriculum, embedding them in specialist training and health professionals completing modules as part of continuous professional development). Learnings from this study will inform ongoing improvements to the training. Future research should explore the extent to which participants apply the training in their clinical practice and whether training has a measurable impact on their decision making and quality of end-of-life care.

## Data Availability

As data collection is ongoing, data from the study are not available. However, additional information regarding the findings presented can be requested from the corresponding author.

## References

[1] White B, Willmott L, White B, Willmott L (2021). End-of-life law reform. International perspectives on end-of-life law reform: politics, persuasion and persistence.

[2] White B, Willmott L, Trowse P (2011). The legal role of medical professionals in decisions to withhold or withdraw life-sustaining treatment: part 1 (New South Wales). J Law Med.

[3] White B, Willmott L, Then S-N, White B, McDonald F, Willmott L (2018). Withholding and withdrawing life-sustaining medical treatment. Health Law in Australia.

[4] White B, Willmott L, Then S-N, White B, McDonald F, Willmott L (2018). Adults who lack capacity: substitute decision-making. Health Law in Australia.

[5] White BP, Willmott L, Feeney R (2021). Limitations in health professionals’ knowledge of end-of-life law: a cross-sectional survey. BMJ Support Palliat Care.

[6] Curnow K (2016). End-of-life decision-making in a health services setting: an access to justice lens. J Law Med.

[7] White BP, Willmott L, Close E (2022). Better regulation of end-of-life care: a call for a holistic approach. J Bioeth Inq.

[8] Sellars M, White B, Yates P (2022). Knowledge of end-of‐life law: a cross‐sectional survey of general practitioners working in aged care. Australas J Ageing.

[9] White B, Willmott L, Cartwright C (2014). Doctors’ knowledge of the law on withholding and withdrawing life-sustaining medical treatment. Med J Aust.

[10] Willmott L, White B, Yates P (2020). Nurses’ knowledge of law at the end of life and implications for practice: a qualitative study. Palliat Med.

[11] Wong AK, Carey SE, Kenner DJ (2021). Can hospital doctors provide quality palliative care informed by end-of-life care legislation: an Australian perspective. Arch Med Health Sci.

[12] Parker M, Willmott L, White B (2015). Medical education and law: withholding/withdrawing treatment from adults without capacity. Intern Med J.

[13] Parker M, Willmott L, White B (2018). Law as clinical evidence: a new constitutive model of medical education and decision-making. J Bioeth Inq.

[14] Morgan DD, Rawlings D, Moores CJ (2019). The changing nature of palliative care: implications for allied health professionals’ educational and training needs. Healthcare.

[15] Donovan LA, Slater PJ, Baggio SJ (2019). Perspectives of health professionals and educators on the outcomes of a national education project in pediatric palliative care: the quality of care collaborative Australia. Adv Med Educ Pract.

[16] Slater PJ, Herbert AR, Baggio SJ (2018). Evaluating the impact of national education in pediatric palliative care: the quality of care collaborative Australia. Adv Med Educ Pract.

[17] Kelley LT, Coderre-Ball AM, Dalgarno N (2020). Continuing professional development for primary care providers in palliative and end-of-life care: a systematic review. J Palliat Care Med.

[18] Donne J, Odrowaz T, Pike S (2019). Teaching palliative care to health professional students: a systematic review and meta-analysis of randomized controlled trials. Am J Hosp Palliat Care.

[19] Hökkä M, Rajala M, Kaakinen P (2022). The effect of teaching methods in palliative care education for undergraduate nursing and medical students: a systematic review. Int J Palliat Nurs.

[20] Li W, Chhabra J, Singh S (2021). Palliative care education and its effectiveness: a systematic review. Public Health.

[21] Rawlings D, Yin H, Devery K (2020). End-of-life care in acute hospitals: practice change reported by health professionals following online education. Healthcare.

[22] Hutchinson C, Tieman J, Devery K (2018). Evaluation of a toolkit resource package to support positive workplace behaviours in relation to quality end-of-life care in Australian hospitals. BMJ Open Qual.

[23] Carter AJE, Arab M, Harrison M (2019). Paramedics providing palliative care at home: a mixed-methods exploration of patient and family satisfaction and paramedic comfort and confidence. CJEM.

[24] Evans JM, Mackinnon M, Pereira J (2021). Building capacity for palliative care delivery in primary care settings: mixed-methods evaluation of the integrate project. Can Fam Physician.

[25] Nagarajan SV, Lewis V, Halcomb E (2022). Barriers and facilitators to nurse-led advance care planning and palliative care practice change in primary healthcare: a qualitative study. Aust J Prim Health.

[26] Pereira J, Meadows L, Kljujic D (2022). Do learners implement what they learn? Commitment-to-change following an interprofessional palliative care course. Palliat Med.

[27] Seow H, Bainbridge D, Stajduhar K (2023). Building palliative care capacity for generalist providers in the community: results from the CAPACITI pilot education program. Am J Hosp Palliat Care.

[28] Slater PJ, Osborne CJ, Herbert AR (2021). Ongoing value and practice improvement outcomes from pediatric palliative care education: the quality of Care Collaborative Australia. Adv Med Educ Pract.

[29] Selman L, Robinson V, Klass L (2016). Improving confidence and competence of healthcare professionals in end-of-life care: an evaluation of the ‘transforming end of life care’course at an acute hospital trust. BMJ Support Palliat Care.

[30] Schwill S, Reith D, Walter T (2020). How to ensure basic competencies in end of life care–a mixed methods study with post-graduate trainees in primary care in Germany. BMC Palliat Care.

[31] Glover TL, Garvan C, Nealis RM (2017). Improving end-of-life care knowledge among senior baccalaureate nursing students. Am J Hosp Palliat Care.

[32] Craig DP, Thompson F (2020). Clinicians’ consent law knowledge: the case for education. Focus Health Prof Educ.

[33] Evans CJ, Ison L, Ellis-Smith C (2019). Service delivery models to maximize quality of life for older people at the end of life: a rapid review. Milbank Q.

[34] White BP, Willmott L, Yates P, et al. End of life law for clinicians [online]. 2019. www.ellc.edu.au . Accessed 2 Nov 2022.

[35] White BP, Willmott L, Neller P. End of life law in Australia [online]. 2019. https://end-of-life.qut.edu.au Accessed 2 Nov 2022.

[36] Bogo M (2015). Field education for clinical social work practice: best practices and contemporary challenges. Clin Soc Work J.

